# Effects of aerobic, strength, and combined training during pregnancy in the blood pressure: A systematic review and meta-analysis

**DOI:** 10.3389/fphys.2022.916724

**Published:** 2022-08-30

**Authors:** Marcelo Corso, Bianca Miarka, Tiago Figueiredo, Nicola Bragazzi, Danilo Carvalho, Ingrid Dias

**Affiliations:** ^1^ Physical Education Postgraduate Program, School of Physical Education and Sports, Federal University of Rio de Janeiro, Rio de Janeiro, Brazil; ^2^ Physical Education Program, Estácio de Sá University, Rio de Janeiro, Brazil; ^3^ Laboratory for Industrial and Applied Mathematics, Department of Mathematics and Statistics, York University, Toronto, ON, Canada

**Keywords:** gestational complications, pregnancy-induced hypertension, physical therapy, high blood pressure, exercise physiology

## Abstract

Gestational hypertension can lead to fetal complications, and, if untreated, high blood pressure during pregnancy may cause eclampsia and even death in the mother and fetus. Exercise is a strategy for preventing blood pressure disorders. There is little knowledge about the physiological impacts of different physical types of training on blood pressure during pregnancy. For that, this meta-analysis aimed to compare the effects of different physical exercise modalities (i.e., aerobic training—AT, strength training—ST, and combined training—AT + ST) on systolic blood pressure (SBP) and diastolic blood pressure (DBP) of pregnant women. A search was performed on PUBMED, LILACS, CINAHL, Sport discus, EMBASE, SCOPUS, and Cochrane Central Register of Controlled Trials to identify researchers. From 3,450 studies, 20 and 19 were included in the qualitative and quantitative analyses. AT studies presented a medium effect size (ES) on SBP [−0.29 (−2.95 to 2.36) *p* = 0.83], with substantial heterogeneity (I^2^ = 64%), and had a large impact on DBP [−1.34 (−2.98 to 0.30) *p* = 0.11], with moderate heterogeneity (I^2^ = 30%). ST researchers showed a large ES on SBP [−1.09 (−3.66 to 1.49) *p* = 0.41], with a reduced heterogeneity (I^2^ = 0%), and a medium ES on DBP [−0.26 (−2.77 to 2.19) *p* = 0.83] with moderate heterogeneity (I^2^ = 38%). AT + ST studies had a large ES on SBP [−1.69 (−3.88 to 0.49) *p* = 0.13] and DBP [−01.29 (−2.26 to 0.31) *p* = 0.01] with considerable (I^2^ = 83%) and moderate heterogeneity (I^2^ = 47%), respectively. These findings are essential for developing new research protocols to avoid gestational hypertension and preeclampsia. AT + ST had a large impact on the SBP and DBP reduction; however, there is a need for more similar procedures to reduce heterogeneity between studies, promoting consensual results.

**Systematic Review Registration:** [PROSPERO], identifier [CRD42021256509].

## 1 Introduction

Diastolic blood pressure (DBP) or Systolic Blood pressure (SBP) disorders are the most common cause of health problems during pregnancy (Regitz-Zagrosek et al., 2018; Sutton et al., 2018). Gestational Hypertension, defined as SBP ≥140 mmHg or DBP ≥90 mmHg (Butalia et al., 2018), is the main cause of maternal health problems, such as less blood flow to the placenta, placental abruption, stroke, disseminated intravascular coagulation, future cardiovascular disease by preeclampsia, and multiple organ failure (Ciapponi et al., 2017; Firoz et al., 2014; Liu & Sun, 2019). In addition, SBP and DBP disorders impact fetus risk (intrauterine growth retardation, intrauterine death, and premature birth) (Regitz-Zagrosek et al., 2018). Given the protective effects of regular exercise on the cardiovascular system, physical exercise is considered a safe and effective non-pharmacological strategy to manage blood pressure (BP) disorders in all conditions (American College of Sports Medicine, 2004) and during pregnancy (Butalia et al., 2018). There is evidence regarding the impacts of physical exercise on BP in pregnant women. However, there are only a few systematic reviews with meta-analyses addressing these effects during pregnancy (Magro-Malosso et al., 2017; Du et al., 2019; Syngelaki et al., 2019; Danielli et al., 2022).

Pregnancy involves several cardiovascular adaptations, such as increased heart rate and cardiac output, associated with vasodilatation (Abbas et al., 2005). These responses associated with the secretion of gestational hormones, circulating prostaglandins, and heat produced by the fetus result in oscillations in the BP values characterized by hypotension in the first and second trimesters and a slight increase in the third (Sanghavi & Rutherford, 2014). Despite these responses, there is evidence that women with body mass index ≥25 kg/m2 have demonstrated significantly higher SBP and DBP (Grindheim et al., 2012), and BP-related disorders are a relevant cause of death in pregnant women. They, therefore, should be strongly considered (Say et al., 2014). In this sense, any resource to prevent such responses has a critical relevance, and one strategy is exercise training.

A previous meta-analysis of randomized controlled trials analyzed the effects of aerobic training (AT) during pregnancy on the risk of gestational hypertensive disorders in 5,075 pregnant women. It showed that exercise reduced the risk of BP disorders (Magro-Malosso et al., 2017). AT is prescribed according to the frequency, intensity, time, volume, and pattern (Liguori & ACSM, 2021). In adults, the AT variables were previously studied. It was shown that larger SBP and DBP reductions in hypertensive subjects, shorter exercise program durations with moderate to high intensity, and programs designed from 150 to 210 min per week (Cornelissen & Smart, 2013). Preceding reports showed positive effects of AT during pregnancy, using chronic protocols of eight to 39 weeks with different programs and session times (e.g., 15–30 min cycle ergometer exercises, 60 min walking, 45–60 min swimming or low impact dance exercises) (Bahadoran et al., 2015; Carpenter et al., 2015; Daniel et al., 2015; Guelfi et al., 2016).

Although some blood pressure medications are considered safe during pregnancy, angiotensin-converting enzyme (ACE) inhibitors, angiotensin II receptor blockers, and renin inhibitors must be avoided during pregnancy (Thangaratinam et al., 2012; Vahedian-Azimi et al., 2021; Liabsuetrakul et al., 2022). In many cases, regular physical activity could positively impact health, potentially offering similar effects to some drug treatments in terms of mortality benefits (Kato et al., 2020; Mustata et al., 2004). AT has been shown to decrease resting plasma norepinephrine (Duncan et al., 1985; Batacan et al., 2015) as well as renal sympathetic nerve activity (Grassi et al., 1994) and muscle sympathetic nerve activity (Liu et al., 2012). AT physiological effects include a cardiac output increases during exercise as the heart can pump more blood each beat delivering more blood if required, the oxygen uptake increases with AT as the blood becomes more efficient, and there is more hemoglobin in the blood to extract oxygen from the lungs (Eriksson et al., 1975; Fernandes et al., 2012; McGee et al., 2018; Lee et al., 2020). The Hemoglobin levels increase with AT to try and get more oxygen to the muscles activity (Yuing et al., 2019); AT exercises strengthen the heart, which increases its stroke volume, which means at rest, the heart does not need to beat as often (Hagerman, 1984), AT, therefore, can promote a lower resting heart rate (Shah et al., 2018). AT could lead to the increased size of slow twitch fibers and minimal change to the fast twitch fibers (Steinacker, 1993). This information, combined with mechanisms responsible for changes in arterial blood pressure after AT, appears numerous and has not been clearly defined in pregnancy (Thangaratinam et al., 2012). In addition, the effectiveness of the AT on the BP values during pregnancy needs to be clarified.

Strength training (ST) is also part of a well-rounded program of exercises (American College of Sports Medicine, 2011), and it is strongly recommended for pregnant women (Mottola et al., 2018). Although some investigations have reported a reduction of arterial BP after ST with muscle sympathetic nerve activity adaptations (Ray & Carrasco, 2000) and the consequent effects of reducing norepinephrine on vascular smooth muscle tone (Carter et al., 2003), it is a few studies with pregnant women (O’Connor et al., 2011).

The manipulation of the ST training variables, such as load intensity, the number of sets and repetitions, order of exercises, and rest interval length between sets and exercises, is part of a design ST periodization (American College of Sports Medicine, 2009). One to three sets from 10 to 15 repetitions, 2 min resting between sets and exercises, at 70% of one-repetition maximum, is recommended during pregnancy for different goals, such as reducing the risk of gestational diabetes, preeclampsia, and lower back pain (Schoenfeld, 2011). A previous meta-analysis shows that manipulating the ST variables is associated with different long-term BP responses (MacDonald et al., 2016). Past studies demonstrated isolated ST or combined with AT during pregnancy, using chronic protocols of 12–39 weeks with a frequency of three to five times a week, maintaining a 12 to 15 Borg rating of perceived exertion scale (Fernández-Buhigas et al., 2020; Silva-Jose et al., 2021). However, there is no consensus about the effects of isolated ST or combined with AT (AT + ST) on BP response in pregnant women.

After searching the literature, some meta-analyses were found to assess the risk of developing blood pressure-related disorders in pregnant women (Magro-Malosso et al., 2017; Du et al., 2019; Syngelaki et al., 2019; Danielli et al., 2022). However, none of them analyzed the behavior of systolic or diastolic blood pressure in its quantitative aspects, considering different types of physical exercises. Our study aimed to show the effects of different physical exercise modalities (i.e., aerobic training—AT; strength training—ST; and combined training—AT + ST) on the BP of pregnant women. The hypothesis is that AT + ST is more efficient than AT or ST on the BP control.

## 2 Materials and methods

In order to verify the effects of different physical exercise modalities on the BP of pregnant women, we conducted a meta-analysis according to the Preferred Reporting Items for Systematic Reviews and Meta-analyses (PRISMA) criteria (Page et al., 2021). The following databases were consulted in May 2021: PUBMED, LILACS, Cumulative Index to Nursing and Allied Health Literature (CINAHL), Sport discus *via* EBSCOhost, EMBASE, SCOPUS, and Cochrane Central Register of Controlled Trials. The references list from other relevant reviews and meta-analyses were also consulted.

### 2.1 Registration and protocol

This research was registered in the International Prospective Register of Systematic Reviews—PROSPERO, with the register number CRD42021256509.

### 2.2 Literature search and study selection

The search routine was based on respective Medical Subject Headings (MeSH) descriptors and other keywords related to the topic. A unique key search was built according to a strategy divided into three parts:

✓ The keywords were listed according to the population, intervention, and outcomes.
✓ All keywords were crossed using the Boolean OR.
✓ The keywords from the population, intervention, and outcomes were crossed between them using the Boolean AND.

After that, the following filters were activated according to each database: humans, adults, women, randomized controlled trials, complete text from scientific journals, and English. The following specific keywords were adopted: pregnant women, pregnancy, pregnancies, gestation, pregnants, resistance training, strength training, strength training program, resistance training program, exercise, physical activity, physical exercise, physical exercises, aerobic exercise, aerobic exercises, exercise training, eclampsia, preeclampsia, blood pressure disorders, blood pressure, hypertension, and high blood pressure. There was no restriction to the period of the search.

### 2.3 Data extraction

#### 2.3.1 Selection criteria

All the studies identified in the databases were uploaded to Rayyan Web. Two independent researchers performed double-blinded studies on identification and data extraction on the Rayyan web app. A third researcher resolved discordances if necessary first, duplicates were located and then removed. After that, titles and abstracts were analyzed. The information extracted from the studies was according to the CERT template (Supplementary Table 1) (Slade et al., 2016). If there was not enough information in these sessions to apply the eligibility criteria, the papers were read in full version.

The research papers were considered according to the following inclusion criteria: 1) studies with pregnant women 18 years old and over; 2) interventions with structured (at least one methodological variable controlled) AT, ST with free weights, machines, elastic bands, or bodyweight, or AT + ST in any modality; 3) SBP and DBP measured at the baseline and after intervention during pregnancy; 4) healthy or any type of chronic disease pregnant women, since the BP pre- and post-intervention were available; 5) randomized controlled trials and longitudinal; 6) original articles published in English. For this systematic review and meta-analysis, the following exclusion criteria were adopted: 1) studies with adolescents; 2) interventions of exercises that were not prescribed by a professional or according to their variables (i.e., volume, intensity, type, duration, number of sets, number of repetitions); 3) ST in isokinetic machines; 4) protocol that involved exercises other than AT or ST, such as isolated pelvic floor muscles training, yoga, or stretching in the experimental or control group; 5) studies with animals.

### 2.4 Assessment of risk of bias

Two researchers were involved in the risk of bias and the quality of the study’s analysis. A third researcher resolved discordances if necessary. It was used the TESTEX tool (Smart et al., 2015) to evaluate the study quality in five questions (eligibility criteria, randomization specification, allocation concealment, group similarity at baseline, and blinding of assessor for at least one key outcome) with one point for each question; and the study reporting in other seven questions (outcome measures assessment, intention-to-treat analysis, statistical comparisons reporting, point measures and measures of variability for all reported outcomes, control group monitoring, relative exercise intensity, and other exercise parameters), in a total of 10 points. Considering all the scales, a score of 15 points is possible. The following criteria were used to verify the risk of bias and quality of the studies: high quality and low risk of bias (≥10 points), moderate quality and risk of bias (7–9 points), poor quality and high risk of bias (1–6 points).

### 2.5 Meta-analysis

A meta-analysis was performed to assess the mean difference between experimental and control groups with a 95% Confidence Interval (CI). A random-effects model was used to consider differences in the protocols, instruments measuring BP, type of exercises, and the intent to generalize the results beyond the included studies. The I2 statistic was verified to assess heterogeneity across studies. Two studies presented data with graphics (Baciuk et al., 2008; Melo et al., 2012). The images were printed and uploaded to Webplotdigitizer software (version 4.4, Pacifica, California, United States), and the data were extracted manually. This software was validated previously (Drevon et al., 2017).

## 3 Results

### 3.1 Studies selection

**FIGURE 1:**
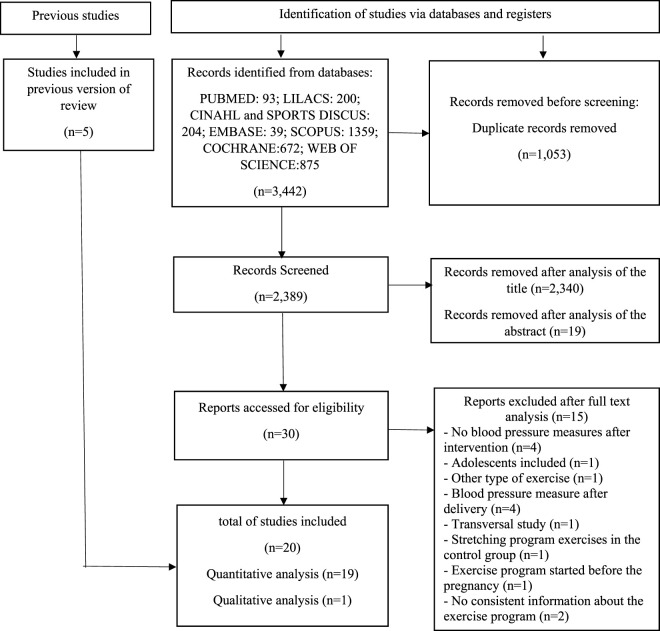
Flow diagram for study selection.

Three thousand four hundred forty-two studies were identified in the database search. One thousand fifty-three duplicates were removed, so 2,389 papers were screened—2,340 after analysis of the title and 19 after reading the abstract. Then, 30 studies were assessed for eligibility and read in full. Fifteen papers were excluded for other reasons: no BP measurements after intervention (*n* = 4); adolescents included (*n* = 1); exercise intervention other than AT or ST (*n* = 1); BP data post-intervention after delivery (*n* = 4); acute intervention (*n* = 1); stretching exercises program in the control group (*n* = 1); exercise program started before the pregnancy (*n* = 1); no consistent information about the exercise program (*n* = 2). Fifteen papers from databases and five from other sources met the inclusion criteria. For the qualitative analysis, 20 studies were included. One did not present the standard deviation, and the quantitative analysis was impossible. The different phases of this study are presented in Figure 1.

### 3.2 Study characteristics and quality

The risk of bias and quality of the studies are in Table 1.

**TABLE 1:** Assessment of risk of bias and quality if the studies.

Study	1	2	3	4	5	6	7	8	9	10	11	12	Score
Baciuk et al. (2008)	1	1	1	1	0	1	1	2	1	0	1	1	11
Bahadoran et al. (2015)	1	0	0	1	0	1	1	2	1	0	1	0	8
Barakat et al. (2012)	1	0	0	1	0	3	1	2	1	0	1	1	11
Barakat et al. (2011)	1	0	0	1	0	3	1	2	1	0	1	1	11
Barakat et al. (2014b)	1	0	0	1	0	3	1	2	1	0	1	1	11
Barakat et al. (2014a)	1	0	0	1	0	1	1	2	1	0	1	1	9
Carpenter et al. (2015)	1	0	0	1	0	1	1	2	1	0	1	1	9
Daniel et al. (2015)	1	1	1	1	0	1	1	2	1	0	1	1	11
Fernández-Buhigas et al. (2020)	1	0	0	1	0	2	1	2	1	1	1	0	10
Garnæs et al. (2016)	1	1	1	1	1	2	1	2	1	1	1	1	14
Guelfi et al. (2016)	1	1	1	1	0	3	1	2	1	0	1	1	13
Haakstad et al. (2016)	1	1	1	0	1	3	1	2	1	0	1	1	13
Melo et al. (2012)	1	1	1	1	1	3	1	2	0	0	1	1	13
O’Connor et al. (2011)	1	0	0	0	0	2	1	0	1	0	1	1	7
Perales et al. (2016)	1	1	0	1	1	3	1	2	1	0	1	1	13
Perales et al. (2020)	1	1	0	1	1	3	1	2	1	0	1	0	12
Ramírez-Vélez et al. (2011)	1	1	0	1	1	2	1	2	1	0	1	0	11
Seneviratne et al. (2016)	1	1	1	1	1	2	1	2	1	0	1	1	13
Silva-Jose et al. (2021)	1	1	1	1	1	2	1	2	1	0	1	1	13
Stutzman et al. (2010)	1	0	0	1	0	1	1	2	1	0	1	1	9

#### 3.2.1 Aerobic training

Eight studies evaluated the SBP and DBP after AT during pregnancy (Baciuk et al., 2008; Stutzman et al., 2010; Melo et al., 2012; Bahadoran et al., 2015; Carpenter et al., 2015; Daniel et al., 2015; Guelfi et al., 2016; Seneviratne et al., 2016). The training frequency ranged from three (Baciuk et al., 2008; Melo et al., 2012; Daniel et al., 2015; Guelfi et al., 2016) to five sessions per week (Stutzman et al., 2010; Bahadoran et al., 2015; Seneviratne et al., 2016), for less than 20 weeks (Stutzman et al., 2010; Daniel et al., 2015; Guelfi et al., 2016; Seneviratne et al., 2016), 20 weeks (Bahadoran et al., 2015), or more than 20 weeks (Baciuk et al., 2008; Melo et al., 2012; Carpenter et al., 2015), in moderate (Stutzman et al., 2010; Daniel et al., 2015; Seneviratne et al., 2016) or intense exercises (Baciuk et al., 2008; Guelfi et al., 2016). The session’s duration was less than 45 min (Melo et al., 2012; Bahadoran et al., 2015; Seneviratne et al., 2016), 45–60 min (Baciuk et al., 2008; Stutzman et al., 2010; Melo et al., 2012; Daniel et al., 2015; Guelfi et al., 2016), and more than 60 min (Carpenter et al., 2015). For the SBP, one study presented a significant increase in the experimental group (EG) (Baciuk et al., 2008) and another study in the control group (CG) (Daniel et al., 2015) compared to baseline. Two papers showed significant reductions in the EG compared to baseline (Daniel et al., 2015; Guelfi et al., 2016). Four studies did not show significant differences between groups or compared to baseline (Melo et al., 2012; Bahadoran et al., 2015; Carpenter et al., 2015; Seneviratne et al., 2016). For the DBP, most of the studies did not show significant differences between EG and CG (Baciuk et al., 2008; Melo et al., 2012; Bahadoran et al., 2015; Carpenter et al., 2015). Two papers showed significant reductions in EG compared to baseline (Daniel et al., 2015; Guelfi et al., 2016), and one study showed a significant increase in the CG compared to baseline (Daniel et al., 2015).

#### 3.2.2 Strength training

Only two studies evaluated the BP responses after ST (O’Connor et al., 2011; Barakat et al., 2014a). Each training program presented the following characteristics: 30 weeks of training, three sessions per week with 55–60-min of duration, performing one set of 10–12 repetitions in 11 exercises (Barakat et al., 2014b); 12 weeks of training, three sessions per week with 45-min of duration, performing two sets of 15 repetitions, with 1 and 2 min between sets and exercises (O’Connor et al., 2011). Booth studies did not show significant differences between the EG and CG or compared to baseline on the SBP and DBP.

#### 3.2.3 Combined training

Most of the studies included (*n* = 10) evaluated the effects of the AT + ST on the BP during pregnancy (Barakat et al., 2011; Ramírez-Vélez et al., 2011; Barakat et al., 2012; Barakat et al., 2014a; Garnæs et al., 2016; Haakstad et al., 2016; Perales et al., 2016, 2020; Fernández-Buhigas et al., 2020; Silva-Jose et al., 2021). The exercise program duration was less than 20 weeks (Ramírez-Vélez et al., 2011; Barakat et al., 2014b) or over (Barakat et al., 2011; Barakat et al., 2012; Garnæs et al., 2016; Perales et al., 2016, 2020; Fernández-Buhigas et al., 2020). The frequency was five sessions per week in one study (Garnæs et al., 2016), and three sessions per week in the others (Barakat et al., 2011; Ramírez-Vélez et al., 2011; Barakat et al., 2012; Barakat et al., 2014a; Perales et al., 2016; Fernández-Buhigas et al., 2020). It was not clear the duration and frequency in one study (Haakstad et al., 2016). The intensity of the AT was moderate (Barakat et al., 2014b; Haakstad et al., 2016; Perales et al., 2016, 2020; Fernández-Buhigas et al., 2020; Silva-Jose et al., 2021) or intense (Barakat et al., 2011; Ramírez-Vélez et al., 2011; Barakat et al., 2012; Garnæs et al., 2016) in sessions with less than 45-min (Barakat et al., 2011, 2012), or ranged between 45 and 60-min (Ramírez-Vélez et al., 2011; Barakat et al., 2014a; Garnæs et al., 2016; Haakstad et al., 2016; Perales et al., 2016, 2020; Fernández-Buhigas et al., 2020). The ST was performed with one (Barakat et al., 2011, Barakat et al., 2012; Barakat et al., 2014b) or multiple sets (Garnæs et al., 2016; Haakstad et al., 2016; Perales et al., 2016; Silva-Jose et al., 2021) of 10 repetitions (Barakat et al., 2014a; Garnæs et al., 2016) or more (Barakat et al., 2011; Barakat et al., 2012; Haakstad et al., 2016; Perales et al., 2016; Silva-Jose et al., 2021). The number of sets and repetitions was not clear in one study (Perales et al., 2020). A significant increase on SBP were observed in the EG in one study (Perales et al., 2016) compared to baseline. The SBP decreased significantly in the EG compared to CG at post intervention in three studies (Garnæs et al., 2016; Haakstad et al., 2016; Perales et al., 2020). Six studies did not show significant differences between groups or compared to baseline on SBP (Barakat et al., 2011, 2012; Ramírez-Vélez et al., 2011; Barakat et al., 2014b; Fernández-Buhigas et al., 2020; Silva-Jose et al., 2021). For the DBP, most of the studies did not show significant differences between the groups or compared to baseline (Barakat et al., 2011; Ramírez-Vélez et al., 2011; Barakat et al., 2012; Barakat et al., 2014a; Garnæs et al., 2016; Perales et al., 2016; Fernández-Buhigas et al., 2020; Perales et al., 2020; Silva-Jose et al., 2021), and one study showed a significant decrease in EG compared to CG at post intervention (Haakstad et al., 2016). The studies characteristics are presented in Table 2.

**TABLE 2:** Description of the studies included in the systematic review.

Study	Participants	BP measurement	Type	Duration	Protocol	SBP	DBP
Aerobic training							
Baciuk et al. (2008)	Less than 20 weeks. of pregnancy, single fetus, no gestational risks EG: *n* = 34 (25.8 ± 4.6 y.) CG: *n* = 37 (24.4 ± 5.8 y.)	Auscultation	AT	20 weeks of pregnancy until to delivery	Water aerobics, duration:50-min, 3 sessions/w., int: 70% HRmax	↑ in EG compared to pre	→
Bahadoran et al. (2015)	First pregnancy, 18 to 22 weeks. of pregnancy, 19–35 years, BMI <30 kg/m2. EG: n = 20 (26.1 ± 3.27 y.) CG: n = 59 (27.0 ± 3.57 y.)	Not reported	AT	20 weeks	Walking 3 to 5 sessions/w., duration: 30–45-min	→	→
Carpenter et al. (2015)	Healthy women, 18 years or over, no complications. EG: *n* = 16 (19–40 + y.) CG: *n* = 34 (19–39 y.)	Hemodynamic monitor	AT (land and water exercises)	20 weeks of pregnancy until to delivery	Land: 18-min of cycling (3-min of wp, 15-min of continuous cycling increasing int: every 2-min until HR target–not published), 10-min of stretching and toning exercises; Water: 10-min wp, 30-min light to moderate int exercises	→	→
Daniel et al. (2015)	Multiparous and nulliparous, with gestational diabetes, normotensive, and sedentary EG: *n* = 15 (32 ± 3.42) CG: *n* = 15 (32.93 ± 4.61)	Not reported	AT	8 weeks	Freq: 2–3 sessions/w.; duration: 45–60-min; wp: 10-min, aerobic dance: 35-min; stretching: 10-min; int: moderate (RPE 12–14)	↓ in EG compared to baseline. ↑ in CG compared to baseline	↓ in EG compared to baseline. ↑ in CG compared to baseline
Guelfi et al. (2016)	Less than 14 weeks. of gestation, >18 y., previous pregnancy with gestational diabetes, BMI between 30 and 35 EG: n = 81 (33.6 ± 4.1) CG: n = 76 (33.8 ± 3.9)	Not reported	AT	14 weeks	Cycle ergometer; freq: 3 sessions/w.; session: 5-min of wp (55%–65% HRmax, RPE 9–11), 5-min (65%–75% HRmax, RPE 12–13 + 5 min of interval. 2 types of intervals: one increasing pedaling rate for 15 s, and the other with an increase in cycling resistance for 30 s (75%–85% HRmax, RPE 14–16) repeated every 2 min; 5-min of Cool-down like wp. Session duration: increased from 20 to 60-min during the program	↓ in EG and CG at pos compared to baseline	↓ in EG and CG at pos compared to baseline
Melo et al. (2012)	Healthy women, sedentary, 13 week. or less of pregnancy, single fetus	Auscultation	AT	13 or 20 weeks of pregnancy until to delivery	Freq: 3 sessions/w.; wp, duration: 15-min of walking and increasing according to the physical capacity, int: 60%–80% HRmax RPE 12–16	→	→
Seneviratne et al. (2016)	18 to 40 years, BMI >25, less than 20 weeks. of pregnancy EG: *n* = 37 (31.6 ± 4.6 y.) CG: *n* = 37 (31.1 ± 5.1 y.)	Oscillometric	AT	20–35 weeks of pregnancy	Exercise on bicycle, int: 40%–59% VO2 reserve; wp: 5-min, cycle: 15–30-min, cool-down: 5-min, freq: 3 to 5 sessions/w	→	→
Stutzman et al. (2010)	20 week. of pregnancy, singleton pregnancy, sedentary, healthy; normal weight (EG1 and CG1); overweight (EG2 and CG1) EG1: n-5 (28.8 ± 6.9 y.) CG1: *n* = 5 (25.8 ± 3.0 y.) EG2: *n* = 6 (28.8 ± 6.9 y.) CG2: *n* = 6 (26.2 ± 6.9 y.)	Photoplethysmography	AT	16 weeks	Walking program; int: low (HRR <40%, RPE 11–13); freq: 5 sessions/w., distance: 0,6 Km to 3.0 Km; wp: 5–10-min and cooldown: (5–10-min) + stretching and range of motion exercises	↑ in CG2 compared to baseline	↑ in CG2 compared to baseline
Strength training							
Barakat et al. (2014b)	Healthy women with no complications on pregnancy EG: *n* = 138 (31.4 ± 3.2 y.) CG: n = 152 (31.7 ± 4.5)	Not reported	ST	8–10 weeks until 38–39 weeks of pregnancy	Duration: 55–60-min, freq: 3 sessions/w., int: 60–75% HRmax estimated by 220-age; wp: 10-min; CORE exercises: 35-min (1 set, 10–12 reps, 11 exercises; Cool-down: 10-min pelvic floor and relaxation	→	→
O’Connor et al. (2011)	Low risk for pregnancy related complications, 21–25 week of pregnancy, with back pain or back pain history. EG: *n* = 32 (29 ± 4 y.)	Auscultation	ST	12 weeks	Session duration: 45-min. Wp: 5-min walking; 6 exercises, 2 sets, 15 reps, velocity 2/2 s, 1-min of interval between sets and 2-min between exercises, low to moderate int (RPE 6–20)	→	→
Combined training							
Barakat et al. (2011)	23 to 38 year., no complications, healthy EG: *n* = 34 (31 ± 3 y.) CG: *n* = 33 (31 ± 3 y.)	Not reported	AT + ST	6–9 weeks of pregnancy until to delivery	Duration: 35–45-min; freq: 3 sessions/w.; int: 70% HRmax 220-age; 25-min wp (7–8-min of walking/stretching); CORE: 25 min (1 set, 10–12 reps, 11 exercises); cool-down (7–8-min of relaxing, pelvic floor exercises); Dance: 1 session/w	→	→
Barakat et al. (2012)	Healthy women, uncomplicated pregnancy, and singleton pregnancy EG: *n* = 40 (32 ± 4) CG: *n* = 43 (31 ± 3)	Not reported	AT + ST	6–9 weeks of pregnancy until to delivery	Duration: 35–45-min; freq: 3 sessions/w (2 on land/1 in water); int: 70% HRmax 220-age. Land: Wp: 7–8-min; CORE: 25-min, 1 set, 10–12 reps, 11 exercises; Cool-down: 7–8-min; dance: 1 session/w.; Water: swimming and aquatic exercises	→	→
Barakat et al. (2014a)	Women with no contraindications to exercise, 6–7 weeks of pregnancy. EG: *n* = 107 (31.57 ± 3.87 y.) CG: *n* = 93 (31.51 ± 3.92 y.)	Not reported	AT + ST	12 weeks	Duration: 55–60-min; freq: 3 session/w., wp: 5-min of walking and stretching; 30-min of dance and ST; 20-min to balance and pelvic floor exercises; light to moderate exercises (55%–60% HRmax or RPE (12–13)	→	→
Fernández-Buhigas et al. (2020)	No complication, <16 weeks of pregnancy, no exercise for more than 30-min 3 days/w. EG: *n* = 41 (33.17 ± 3.19) CG: *n* = 51 (32.63 ± 4.66)	Oscillometric	AT + ST	12–15 weeks of pregnancy until to delivery	Duration: 60-min; freq: 3 sessions/w.; wp: 10-min; AT: 25-min (55%–60% HRmax, Borg 12–14), ST: 10-min; 15-min coordination exercises, pelvic floor exercises, and stretching	→	→
Garnaes et al. (2016)	BMI ≥28, ≥18 year., one fetus, and healthy women. EG: *n* = 38 (31.3 ± 3.8 y.) CG: *n* = 36 (31.4 ± 4.7 y.)	Oscillometric	AT + ST	12–18 weeks of pregnancy until to delivery	Session duration: 60-min, 3 sessions/w.; AT: Walking/jogging (35-min); Int: 80% maximal capacity (RPE 12–15); ST: (25-min) for large muscles and pelvic floor; 3 sets, 10 reps, 1 min. At home: 50-min at least once a week (35-min AT, 15-min ST) and daily pelvic floor exercises	↓ in EG compared to CG at post	→
Haakstad et al. (2016)	Nuliparous, healthy pregnancy, no exercise more than once a week EG: *n* = 35 (31.5 ± 3.1 y.) CG: *n* = 26 (29.4 ± 3.8 y.)	Auscultation	AT + ST	Second trimester (12–24 weeks) until to last trimester (36–38 weeks)	Duration: 60-min; AT: Dance 35–40-min, at least 2 sessions/w.; Int: RPE 12–14. Advised: 30-min (walking or bicycle or water exercises); ST: duration: 15-min; 3 sets, 12–15 reps, 4 exercises; Cool-down: 5-min	↓ in EG compared to CG at post	↓ in EG compared to CG at post
Perales et al. (2016)	Healthy, no complications, <16 weeks. of pregnancy, no exercise more than 30 min 3 sessions/w. before gestation. EG: *n* = 83 (31 ± 4 y.) CG: *n* = 59 (31 ± 4 y.)	Not reported	AT + ST	9–11 weeks of pregnancy until to delivery	Freq: 3 sessions/w.; duration: 55/60-min; int: 55%–60% HRres; 5–7-min wp; AT: 25/30-min; ST: ∼30-min; 1 set, 15 reps (1° trimester); 2 sets, 15 reps (2° trimester	↑ in EG and CG compared to baseline	→
Perales et al. (2020)	8–45 years, free of contraindications, uncomplicated gestation, EG1: Overall data n = 688 (32 ± 4 y.) CG1: Overall data *n* = 660 (31 ± 4 y.) EG2: Previously active *n* = 117 (32 ± 4 y.) CG2: Previously active *n* = 562 (31 ± 4 y.) EG3 Previously inactive *n* = 571 (32 ± 4 y.) CG3: Previously inactive *n* = 98 (32 ± 5 y.)	Not reported	AT + ST	9 weeks until to 38/39 weeks of pregnancy	Duration: 50–55-min of low-impact dance and stretching. ST: 4 exercises, light loads; pelvic floor training; Int: 60% HRmax determined by 220-age and RPE 10–12	↓ in EC2 compared to EC3 at pos ↓ in EG3 compared to CG3 at post	→
Ramírez-Vélez et al. (2011)	Nuliparous, 16–20 weeks. of pregnancy, healthy. EG: *n* = 24 CG: *n* = 26 19.5 ± 2.3 years	Auscultation	AT + ST	11 weeks	AT: 30-min, 3 sessions/w., int: moderate to vigorous ST: 25-min Cool-down: 5-min	→	→
Silva-Jose et al. (2021)	18–45 years, no complications, no contraindications for exercise. EG: *n* = 31 (32.29 ± 6.36) CG: *n* = 42 (33.93 ± 4.59)	Not reported	AT + ST	∼30 weeks	Freq: 3 sessions/w.; duration: 55–60-min, Moderate int (55%–65% HRR, RPE 12–14); Wp 5–7-min; AT (8–10 min), ST (10–12-min), 2–3 sets, 10–12 reps; Coordination and balance exercises (5–8-min); Pelvic floor exercises (8–10-min); Cool-down (7–8-min)	→	→

w: weeks; y: years; min: minutes; sec: seconds; EG: exercise group; CG: control group; BMI: body mass index; AT: aerobic training; ST: strength training; AT + ST: combined training; HR: heart rate; max: maximum; HRR: heart rate reserve; RPE: the rating of perceived exertion; wp: warm-up; Freq: frequency; Int: intensity; reps: repetitions; ↑: significant increase; ↓: significant decrease; →: no significant statistical difference.

### 3.3 Meta-analysis results

#### 3.3.1 Aerobic training

**FIGURE 2:**
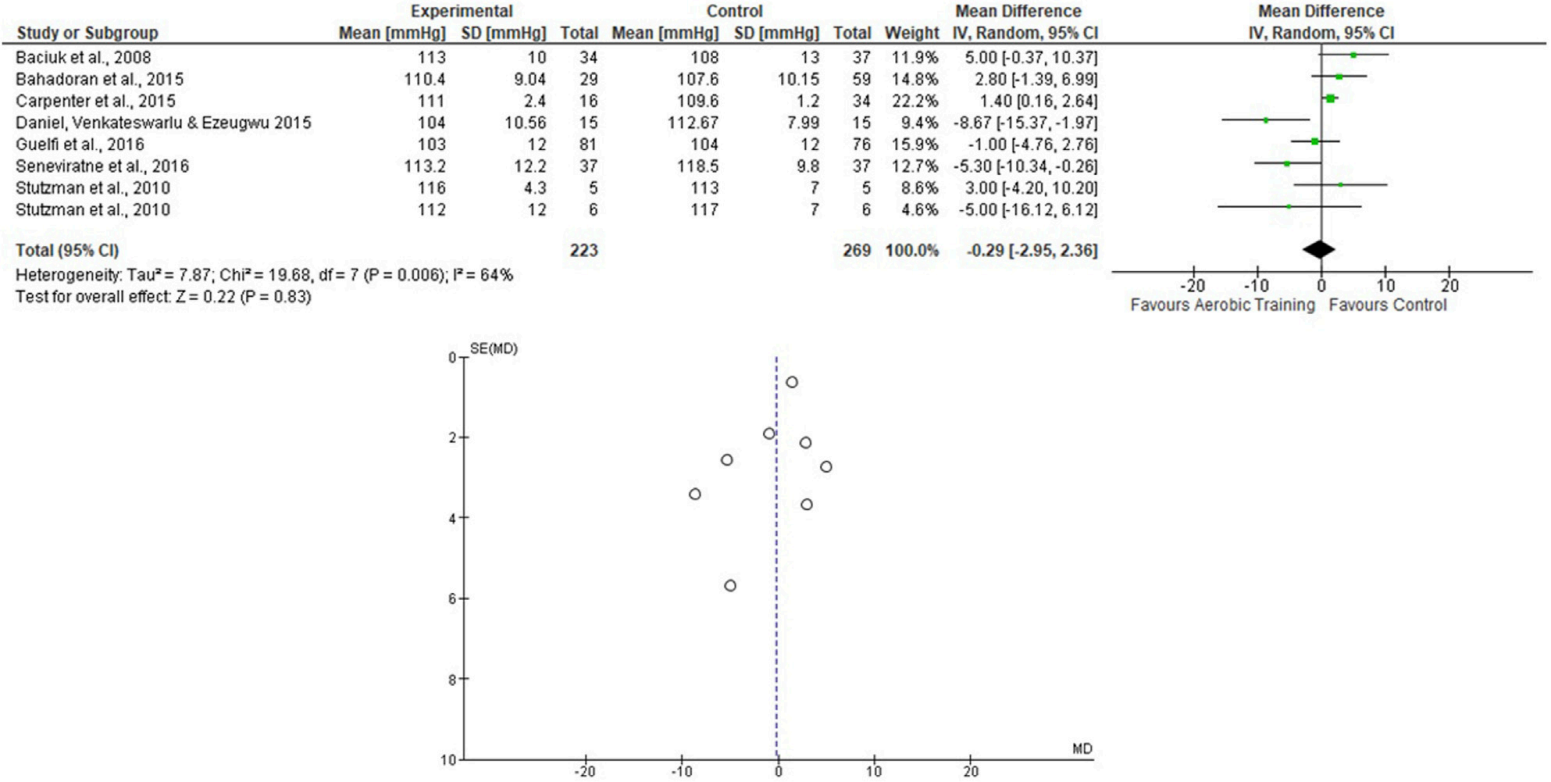
Effects of the AT on SBP in pregnant women and funnel plot for detecting bias and systematic heterogeneity.

**FIGURE 3:**
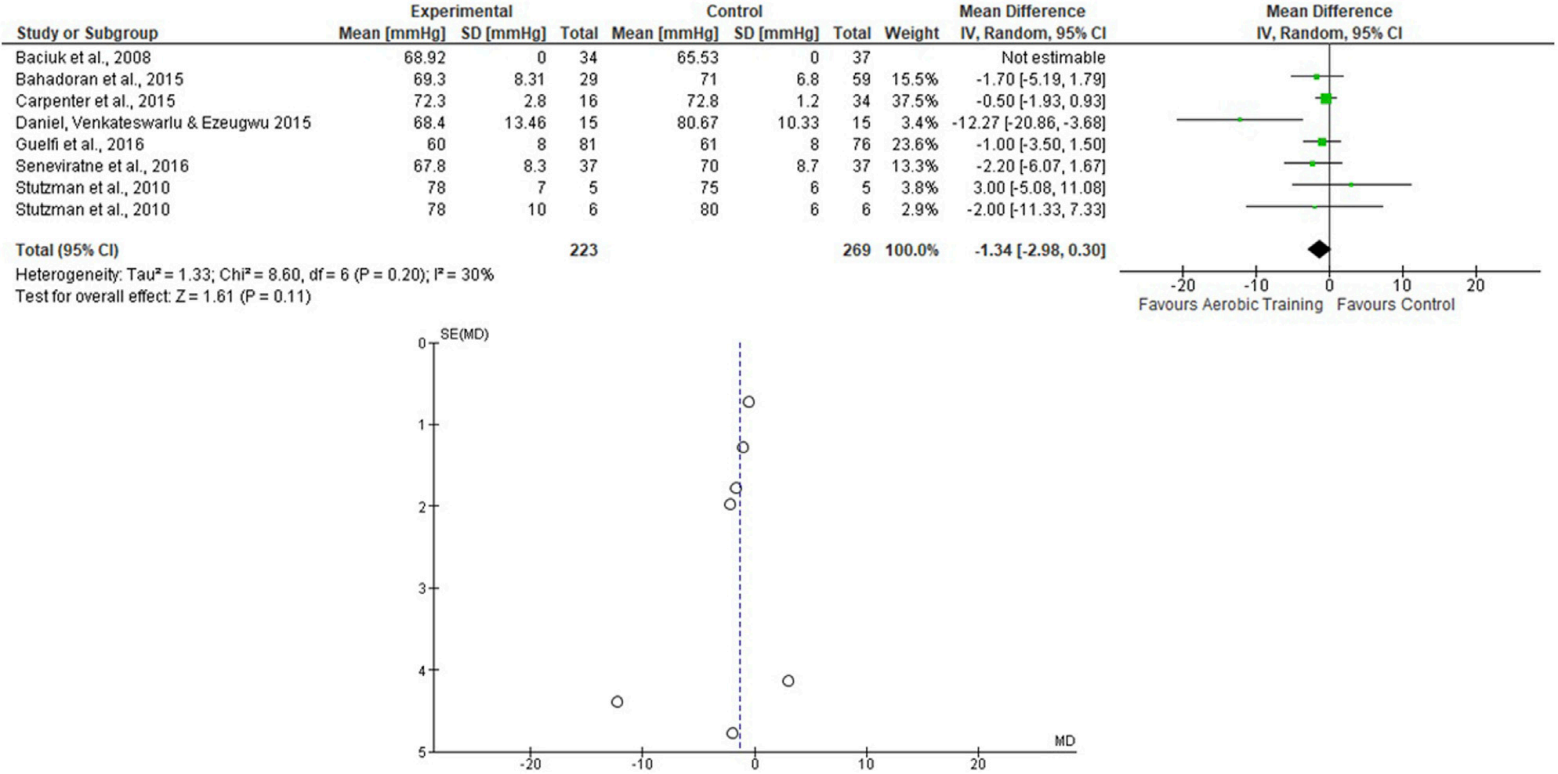
Effects of the AT on DBP in pregnant women and funnel plot for detecting bias and systematic heterogeneity.

For the AT analyses, Stutzman et al. (2010) presented 2 EG and 1 CG, and they were analyzed separately. We assessed 223 participants in the EG and 269 in the CG. The pooled effects of the AT on SBP and DBP are presented in Figures 2, 3, respectively. For the SBP, the heterogeneity was moderate (Tau^2^ = 7.87; Chi^2^ = 19.68; df = 7; *p* = 0.006; I^2^ = 64%) and it was observed a non-significant mean difference (mean difference: −0.29; IC: −2.95–2.36; *p* = 0.83) favoring the EG. For the DBP, the heterogeneity was also moderate (Tau^2^ = 1.33; Chi^2^ = 8.60; df = 6; *p* = 0.20; I^2^ = 30%) and it was observed a non-significant mean difference (mean difference: −1.34; IC: −2.98–0.30; *p* = 0.11) favoring the EG. The effects of the AT on SBP and DBP in pregnant women and the funnel plot for detecting bias and systematic heterogeneity are presented in Figures 2, 3, respectively.

#### 3.3.2 Strength training

**FIGURE 4:**
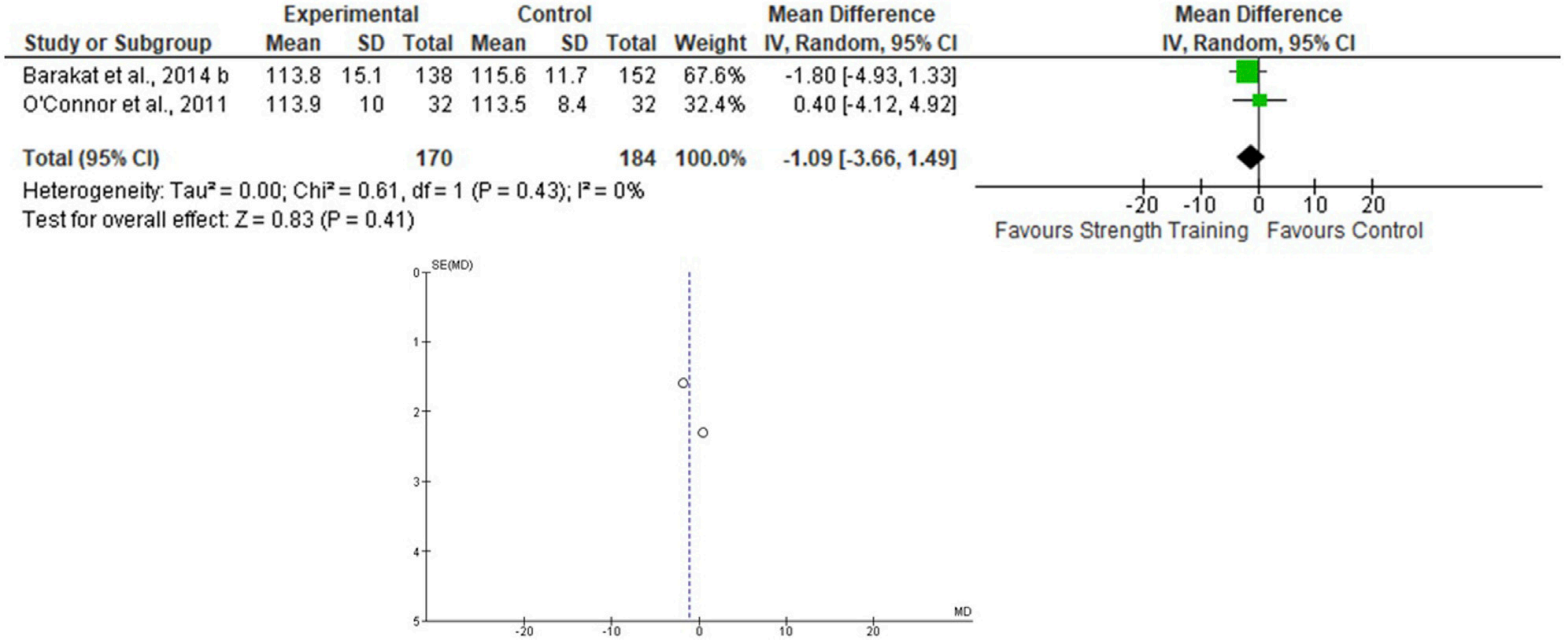
Effects of the ST on SBP in pregnant women and funnel plot for detecting bias and heterogeneity.

**FIGURE 5:**
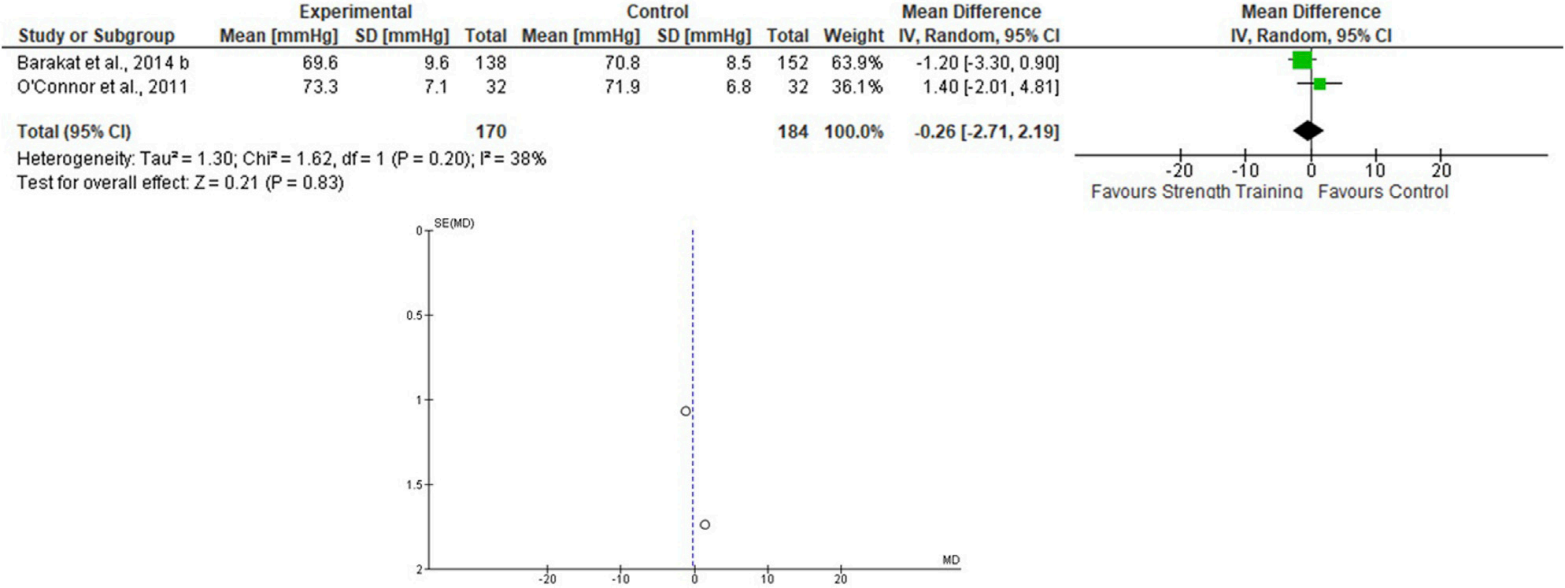
Effects of the ST on SBP in pregnant women and funnel plot for detecting bias and heterogeneity.

Only two studies that assessed the effects of the ST on BP during pregnancy were included (O’Connor et al., 2011; Barakat et al., 2014b), totaling 170 in the EG 184 in the CG. For the SBP, the heterogeneity was low (Tau^2^ = 0.00; Chi^2^ = 0.61; df = 1; *p* = 0.43; I^2^ = 0%) and it was demonstrated a non-significant mean difference (mean difference: −1.09; IC: −3.66–1.49; *p* = 0.41) favoring the training group. For the DBP, the heterogeneity was also low (Tau^2^ = 1.30; Chi^2^ = 1.62; df = 1; *p* = 0.20; I^2^ = 38%) and it was also demonstrated a non-significant mean difference (mean difference: −0.26; IC: −2.71–2.19; *p* = 0.83) favoring the training group. The pooled effects of the ST on SBP and DBP are presented in Figures 4, 5, respectively.

#### 3.3.3 Combined training

**FIGURE 6:**
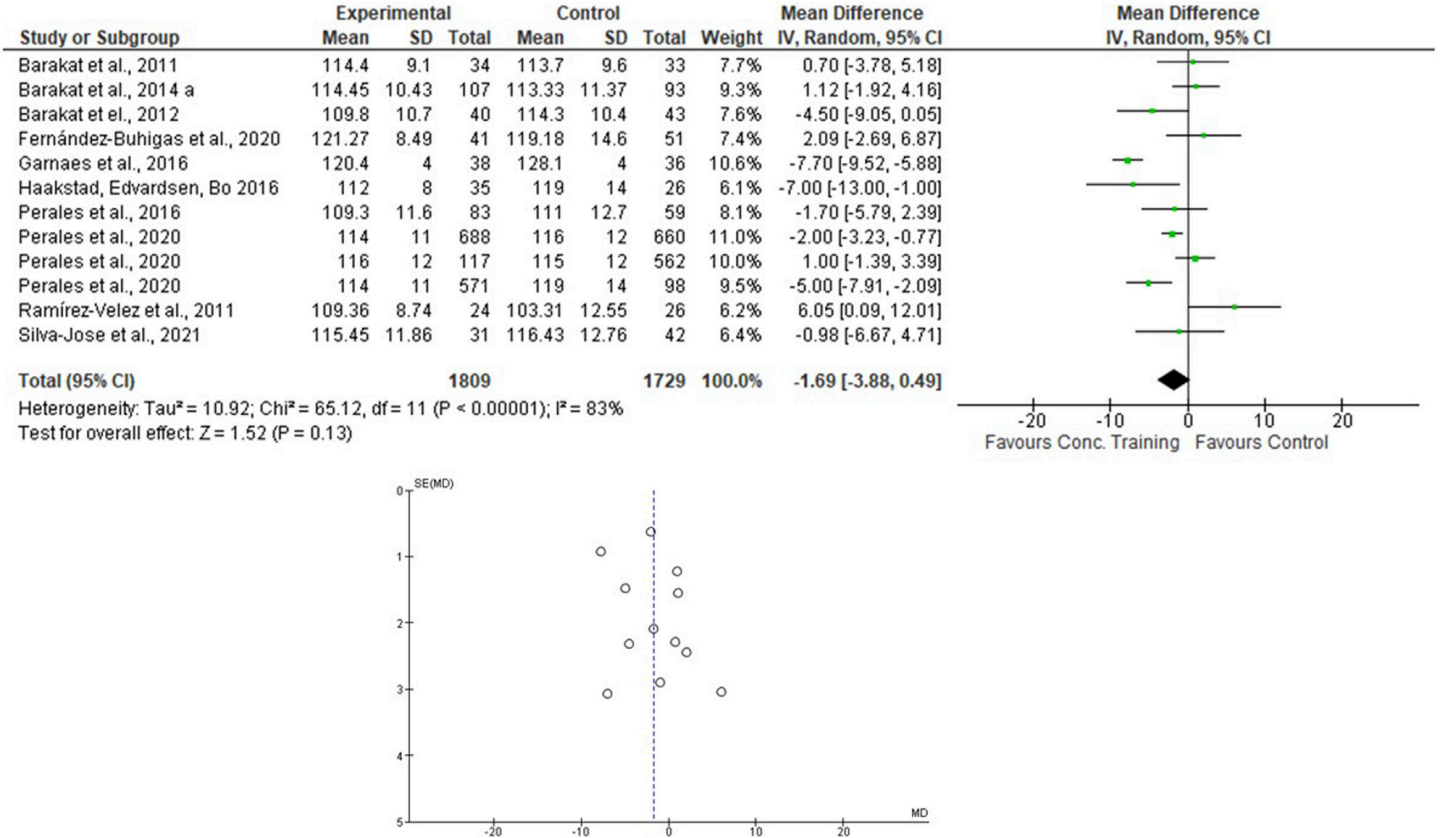
Effects of the AT + ST in pregnant women and funnel plot for detecting bias and heterogeneity.

**FIGURE 7:**
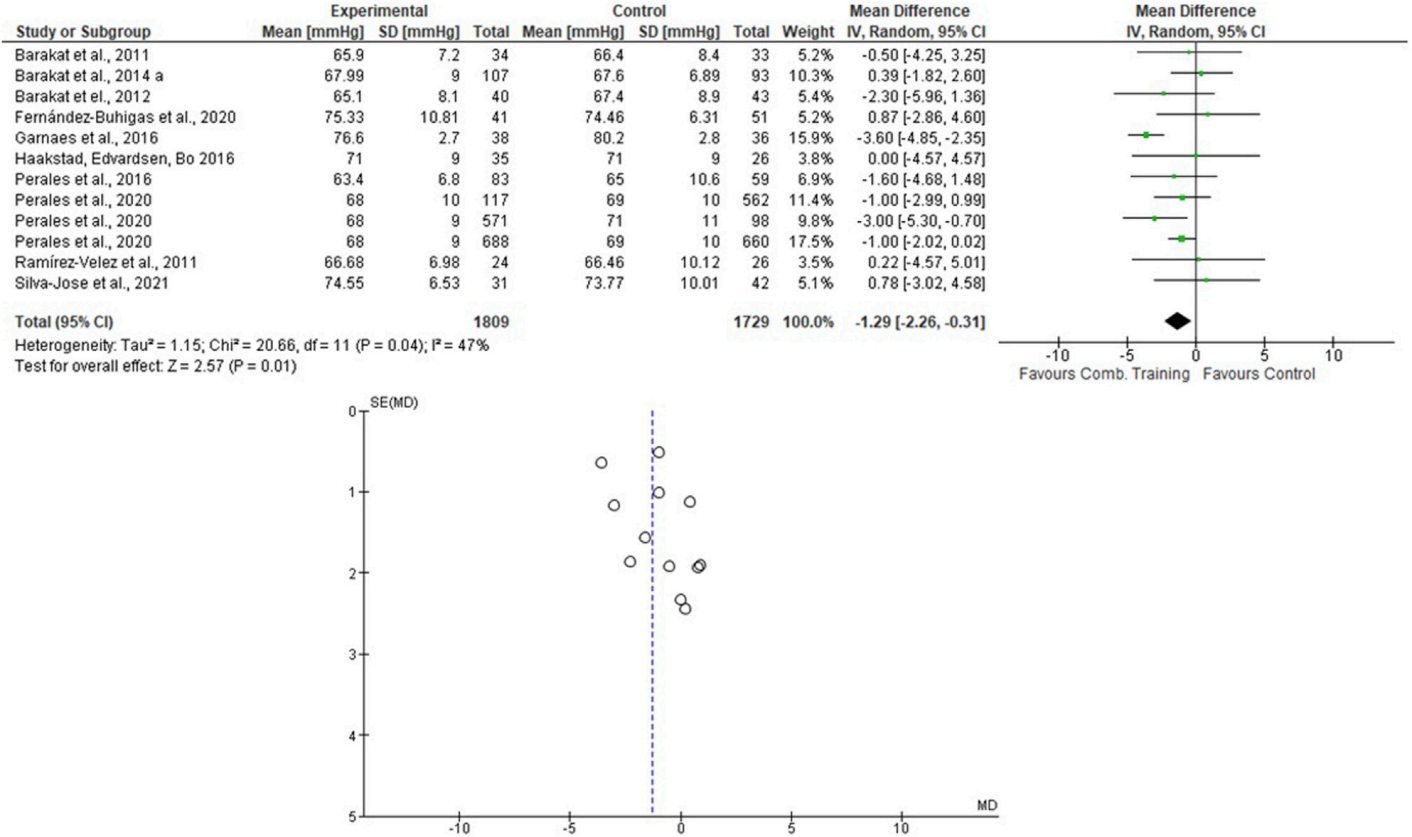
Effects of the AT + ST on DBP in pregnant women and funnel plot for detecting bias and heterogeneity.

For the AT + ST analyses, Perales et al. (2020) presented the results separately. Therefore, this was done in the meta-analysis. One thousand eighty-nine women were allocated in the EG and 1729 in the CG. For the SBP, the heterogeneity was high (Tau^2^ = 10.92; Chi^2^ = 65.12; df = 11; *p* < 0.001; I^2^ = 83%) and it was shown a non-significant mean difference (mean difference: -1.69; IC: -3.88–0.49; *p* = 0.13) favoring the training group. For the DBP, the heterogeneity was moderate (Tau^2^ = 1.15; Chi^2^ = 20.66; df = 11; *p* = 0.04; I^2^ = 47%). The mean difference was significant favoring AT + ST group compared to control (mean difference: −1.29; IC: −2.26 to −0.31; *p* = 0.01). These results are presented in Figures 6, 7.

## 4 Discussion

This systematic review and meta-analysis focused on verifying the effects of different modalities of physical exercises (AT, ST, and AT + ST) on SBP and DBP of pregnant women. The results partially confirmed the hypothesis that the AT + ST is more efficient than AT or ST in the BP control during pregnancy once the effects on SBP were similar and the DBP reduced significantly only after an AT + ST program. The significant findings were as follows:

• The mean difference was significant, with a large effect size favoring the AT + ST group compared to CG for lowering the DBP levels.
• The pooled effects of the AT, ST, and AT + ST demonstrated a non-significant medium/large effect size in SBP in favor of EG compared to CG. The same was observed in the AT + ST on SBP.
• The I^2^ statistic revealed high heterogeneity in the studies that compared the effects of AT and AT + ST on SBP, moderate heterogeneity in the studies that compared the effects of all types of exercises on DBP, and low heterogeneity in the studies that compared the effects of the ST on SBP.

This study showed that the mean difference was significant, favoring the AT + ST group over CG for lowering the DBP levels (see Figure 7). The differences between the activities can explain it. AT + ST is represented by performing AT and ST in a single training session. This condition implies a greater absolute volume of training due to the time in which the skeletal muscles are under tension. For example: among the included studies that evaluated the effects of AT, two of them included stretching exercises in their protocols (Carpenter et al., 2015; Daniel et al., 2015), and one progressed the duration of the exercises (volume) according to the progress of the training program (Guelfi et al., 2016), which, in part, compromised the total duration of the aerobic exercise itself.

According to the literature, training volume is one of the variables that most influence the BP reduction in normotensive and hypertensive subjects (American College of Sports Medicine, 2004; MacDonald et al., 2016; MacDonald & Pescatello, 2019). On the other hand, most of the AT + ST studies showed a session duration ranging from 45 to 60 min. It represents a more excellent training volume. In addition, a meta-analysis has previously demonstrated the maintenance of blood pressure values in SBP and the reduction in DBP after an AT + ST program (Cornelissen & Smart, 2013). Considering that the SBP remained stable and was evidenced in a reduction in DBP, these findings are under the literature, which reaffirms the role of physical exercise in reducing the risk of disorders related to blood pressure in pregnancy (Magro-Malosso et al., 2017; Du et al., 2019; Syngelaki et al., 2019; Danielli et al., 2022). To the best of the authors’ knowledge, there are no meta-analyses that synthesize longitudinal studies that evaluated the impacts of regular physical exercise on BP values during pregnancy. However, Aune et al. (2014) analyzed the dose-response relationships between the level of physical activity and preeclampsia from case-control and cohort studies in 10,317 women. The results showed an inverse association between high physical activity levels and a lower risk of preeclampsia. Although this study included all types of physical activity, the practice of structured AT and ST have been encouraged (Gregg & Ferguson, 2017; Mottola et al., 2018).

The results obtained in the meta-analysis of the present study can be explained by the physiological adaptations that occur in pregnancy and by the physiological adaptations that occur as a result of physical exercise (May, 2015). Pregnancy increases heart rate, stroke volume, and cardiac output and reduces systemic vascular resistance, explained by increases in progesterone, oxide nitric, relaxin, and prostaglandins that result in relaxation of the vasculature (May, 2015; Zeng, Liu & Li, 2017). On the other hand, AT + ST simultaneously results in specific adaptations of AT and ST. Long-term adaptations related to AT by pregnant women are described in the literature. Reductions can explain them in resting heart rate, cardiac output, and greater activation in parasympathetic modulation assessed through heart rate variability (May, 2015). However, the effects of ST or AT + ST on the cardiovascular system of pregnant women need to be better elucidated. However, it is known that the mechanisms that regulate the BP drop after an isolated ST session differs between men and non-pregnant women (Queiroz et al., 2013). While men have a hypotensive effect mediated by a reduction in cardiac output (central adjustment), women have a drop in BP determined by a decrease in systemic vascular resistance (a peripheral mechanism) (Queiroz et al., 2013). Given that DBP is closely related to systemic vascular resistance (Li et al., 2014), it can be speculated that ST in AT + ST sessions plays a crucial role in decreasing DBP, as the acute adaptations described in the literature can influence long-term responses. Despite this evidence, further studies are needed to confirm this narrative.

The present research included protocols ranging from 6 weeks of pregnancy until birth (see table 2), and the pooled effects of the AT, ST, and AT + ST demonstrated a non-significant decrease in SBP in favor of EG compared to CG. The same was observed in the AT + ST on SBP (see Figures 2–6). On the other hand, the current literature presented data that provide a significant clinical effect for these results. Loerup et al. (2019) studied changes in SBP and DBP during pregnancy. The authors included 39 papers and analyzed 124,349 systolic measurements from 36,239 women and 124,291 diastolic measurements from 36,181 women. They demonstrated that the SBP rises approximately 5.6 mmHg between 10 and 40 weeks of gestation, and the DBP is lowest at 21 weeks, rising by 6.9 mmHg by 40 weeks. Therefore, considering these BP fluctuations, maintaining the SBP and DBP is positive for physical exercise. The meta-analysis results confirm these data by Magro-Malosso et al. (2017), which evaluated the effect of AT during pregnancy on the risk of gestational hypertensive disorders in 5,075 women and demonstrated that AT significantly reduced the risk of hypertensive disorders in pregnant women.

The I^2^ statistic revealed high heterogeneity in the studies that compared the effects of AT and AT + ST on SBP (see Figures 2, 6, respectively) and moderate heterogeneity in the studies that compared the effects of all types of exercises on DBP (see Figures 3, 5, 7), and low heterogeneity in the studies that compared the effects of the ST on SBP (see Figure 4). The high heterogeneity observed when SBP was analyzed may be explained by the differences between protocols, such as training frequency, intensity, duration, volume, and modality, which makes the uniformity between protocols complex, mainly the SBP responses, much more sensitive to acute training changes (Hellsten & Nyberg, 2016). For the AT + ST training, the heterogeneity is even more pronounced (see Figure 6). It may be explained by the differences in the exercise protocols, such as the AT, and the interaction between the AT and the ST, which makes the homogeneity even more difficult. The low heterogeneity observed between the studies that evaluated the effects of isolated ST on the BP of pregnant women may be explained by a reduced number of papers included (*n* = 2) (Barakat et al., 2014a; O'Connor et al., 2011).

The practice of physical exercise, especially aerobic training, is recommended for pregnant women (Butalia et al., 2018). However, some absolute and relative contraindications are proposed by recent guidelines (Mottola et al., 2018). Hypertensive disorders include contraindications such as preeclampsia, uncontrolled hypertension, and gestational hypertension. Nevertheless, Meah et al. (2020) proposed a review and reassessment of these contraindications through evidence demonstrating that exercise practice could be safe, even under adverse conditions. Although there is no consensual evidence on how exercise affects BP in pregnant women with hypertensive disorders, it can be speculated that they may benefit from post-exercise hypotension (PEH), a drop in blood pressure a few hours after a single session of AT, ST, or AT + ST (Carpio-Rivera et al., 2016; Casonatto et al., 2016). PEH reduces the time of exposure to high BP, in addition to having a dose-response of the occurrence of PEH and long-term (LIU et al., 2012) and ambulatory blood pressure control (Saco-Ledo et al., 2021). All the participants of the papers included in this study were normotensive. This may explain why the SBP remained stable in the experimental groups in all types of exercise, and the DBP did not change significantly after AT and ST. Individuals classified as pre-hypertensive or hypertensive tend to experience a more significant drop in BP (Cornelissen & Smart, 2013; MacDonald et al., 2016). Regardless of the results obtained, we recommend the practice of AT, ST, and AT + ST according to the evidence presented here (see table 2).

Nevertheless, some gaps in the literature can still be filled, as the included studies presented few details about the ST’s AT + ST protocols. Furthermore, only two studies that evaluated the effects of the isolated ST on BP during pregnancy were included. Therefore, we recommend longitudinal studies with accurate control of training variables, mainly in ST, such as load, total volume, interval length between sets and exercises, exercise selection, range of motion, or training methods. All these variables modify the BP responses (Casonatto et al., 2016; MacDonald et al., 2016). Furthermore, following Meah, Davies, and Davenport (2020), we recommend longitudinal studies aiming to evaluate the effects of different training variables on the BP of pregnant women with hypertensive disorders.

Present research presents limitations. Only two reported that the CG activity was monitored (see table 1, question 10). Studies in table 1 did not demonstrate the estimate of the level of physical activity performed in the daily lives of these pregnant women. Despite a rigorous database search, the data related to ST is limited. Only two studies that assessed BP responses after ST were included. In addition, the ST training variables (i.e., load intensity, number of sets and repetitions, number of exercises, rest interval length between sets and exercises, and mode) during AT + ST protocols were poorly described in the studies. Therefore, it is difficult to interpret the practical applications for AT + ST prescription during pregnancy.

## 5 Conclusion

This systematic review and meta-analyses demonstrated that the structured AT and ST are reliable and safe strategies for maintaining BP at optimal levels during pregnancy. The findings on bias recommend that future studies carry out similar AT, ST, and AT + ST protocols to reduce heterogeneity, verifying more accurate effects. Additionally, the mean difference was significant, favoring the AT + ST group compared to CG for lowering the DBP. These findings are essential for developing practical applications with AT + ST exercises to avoid gestational hypertension and preeclampsia, allowing better guidance in recommendations for physical exercise in this population.

## Data Availability

The original contributions presented in the study are included in the article/Supplementary Material, further inquiries can be directed to the corresponding author.
